# The Neutrophil-to-Lymphocyte Ratio (NLR) as a Potential Predictor in Conization Outcomes for Cervical Cancer

**DOI:** 10.3390/cancers17111856

**Published:** 2025-05-31

**Authors:** Balázs Vida, Emese Keszthelyi, Zsófia Tóth, Lotti Lőczi, Barbara Sebők, Petra Merkely, Balázs Lintner, Ferenc Bánhidy, Attila Keszthelyi, Szabolcs Várbíró, Richárd Tóth, Márton Keszthelyi

**Affiliations:** 1 Department of Obstetrics and Gynecology, Semmelweis University, 1082 Budapest, Hungary; vida.balazs.lajos@semmelweis.hu (B.V.); keszthelyibp@gmail.com (E.K.); toth.zsofia99@stud.semmelweis.hu (Z.T.); keszthelyi.lotti.lucia@semmelweis.hu (L.L.); merkely.petra@gmail.com (P.M.); lintner.balazs.zoltan@semmelweis.hu (B.L.); banhidy.ferenc@semmelweis.hu (F.B.); varbiroszabolcs@gmail.com (S.V.); toth.richard@semmelweis.hu (R.T.); 2 Workgroup of Research Management, Doctoral School, Semmelweis University, 1085 Budapest, Hungary; sebok.barbara23@gmail.com; 3 Department of Urology, Semmelweis University, 1082 Budapest, Hungary; keszthelyi.attila@semmelweis.hu; 4 Department of Obstetrics and Gynecology, University of Szeged, 6725 Szeged, Hungary

**Keywords:** cervical cancer, neutrophil-to-lymphocyte ratio, biomarkers, conization, systemic inflammation, oncology

## Abstract

**Background**: Cervical cancer is a major global health concern, particularly in low-resource settings where access to advanced diagnostics is limited. The neutrophil-to-lymphocyte ratio (NLR), a systemic inflammatory marker, has shown prognostic significance in various malignancies. However, its role in predicting cervical cancer in patients undergoing conization remains unclear. **Objectives**: This study evaluated NLR’s diagnostic and predictive value in identifying malignancy in conization outcomes. **Methods**: This retrospective study analyzed 374 patients from the SCOPE study. Preoperative NLR levels were assessed with conization histopathology outcomes using the Kruskal–Wallis test, Mann–Whitney U test, logistic regression, and receiver operating characteristic (ROC) curve analysis. **Results**: NLR levels were significantly higher in patients with malignant conization outcomes (χ^2^ = 15.988, *p* = 0.001). A cut-off NLR ≥ 2.86 significantly predicted cervical cancer presence (*p* = 0.045). Logistic regression showed that each unit increase in the NLR increased cancer likelihood by 37.2% (*p* = 0.008). ROC analysis demonstrated moderate diagnostic accuracy (AUC = 0.734), with an optimal NLR cut-off of ≥1.865 yielding 87.0% sensitivity and 53.8% specificity. **Conclusions**: Our findings suggest that the NLR may serve as a promising, easily accessible, non-invasive biomarker with modest predictive value for cervical cancer in patients undergoing conization. Future studies should focus on validating the NLR in combination with other biomarkers, such as HPV genotyping and molecular markers, to improve diagnostic accuracy, particularly in low-resource settings.

## Simple Summary

Cervical cancer remains a significant global health burden, particularly in regions with limited access to advanced diagnostic technologies. Identifying reliable and accessible biomarkers is essential for improving early detection. This study investigates the potential role of the neutrophil-to-lymphocyte ratio, a marker derived from routine blood tests, in predicting the presence of cervical cancer among patients undergoing conization. The findings demonstrate that elevated levels of this marker are significantly associated with malignant outcomes, suggesting its utility as a cost-effective, non-invasive predictor of cancer. Incorporating this parameter into preoperative evaluations may enhance clinical decision-making, especially in resource-constrained settings. These results support further integration of systemic inflammatory markers into cervical cancer screening protocols to improve diagnostic accuracy and optimize patient care.

## 1. Introduction

Cervical cancer ranks as the fourth most prevalent cancer among women globally, with the highest incidence occurring between the ages of 30 and 35. In 2022, gynecological malignancies were responsible for an estimated 680,000 deaths worldwide, reflecting a concerning upward trend in incidence [1,2]. Unlike developed countries, cervical cancer is a significant public health issue in developing nations like India, which alone accounts for a quarter of the global burden, where it stands for 6–29% of all female cancer [3,4]. Despite developments in screening methods, the disease is often diagnosed at advanced stages due to inadequate early detection and inconsistent follow-up care. Persistent infection with high-risk human papillomavirus (HPV) is the primary etiological factor, contributing to over 95% of cervical cancer cases [5]. The disease progresses through stages of cervical intraepithelial lesions (CIN1, CIN2, CIN3), which can be identified and treated effectively if detected early [6]. Screening methods like colposcopy and biopsy are vital but often inaccessible.

Loop Electrosurgical Excision Procedure (LEEP) is a common diagnostic and therapeutic tool for managing CIN. It can reveal more advanced lesions than initially predicted by cytology, highlighting limitations in traditional screening methods [7].

The neutrophil-to-lymphocyte ratio (NLR) has gained prominence as a significant prognostic biomarker in assessing gynecological cancers, reflecting the interplay between inflammation and immune response in tumor development [8]. The NLR is calculated from a peripheral blood test and reflects the balance between acute inflammation and the adaptive immune response [9]. It has shown prognostic relevance in oncology and in the field of gynecological cancers, with elevated levels linked to worse outcomes in ovarian, endometrial, and cervical cancers [10,11]. However, its role in predicting malignancy during diagnostic procedures like LEEP conization is not well established. The NLR may also indicate treatment response and aid in identifying high-risk patients requiring intensified management [12,13]. However, variation in cut-off values and reliance on retrospective data pose limitations.

Prognostic markers relate to the course of disease over time, while a *diagnostic* marker helps identify the presence of disease, and predictive markers help forecast the likelihood of a specific outcome, such as the presence of invasive cancer, in a given clinical context [14]. Therefore, our study aims to investigate whether the NLR can serve as a predictive marker for cervical cancer in conization outcomes. We aim to determine a threshold NLR value associated with malignancy and assess its potential integration into preoperative risk stratification.

## 2. Materials and Methods

### 2.1. Patients

This retrospective observational study analyzed a cohort of 374 patients as part of the SCOPE study (Semmelweis University Conization and Inflammation Outcomes with Predictive Evaluation). The research involved an extensive review of medical records spanning from 2021 to 2024. A comprehensive dataset was compiled, incorporating sociodemographic factors, gynecological history, clinical findings, and laboratory results to gain a deeper insight into patient outcomes following conization.

**Figure 1:**
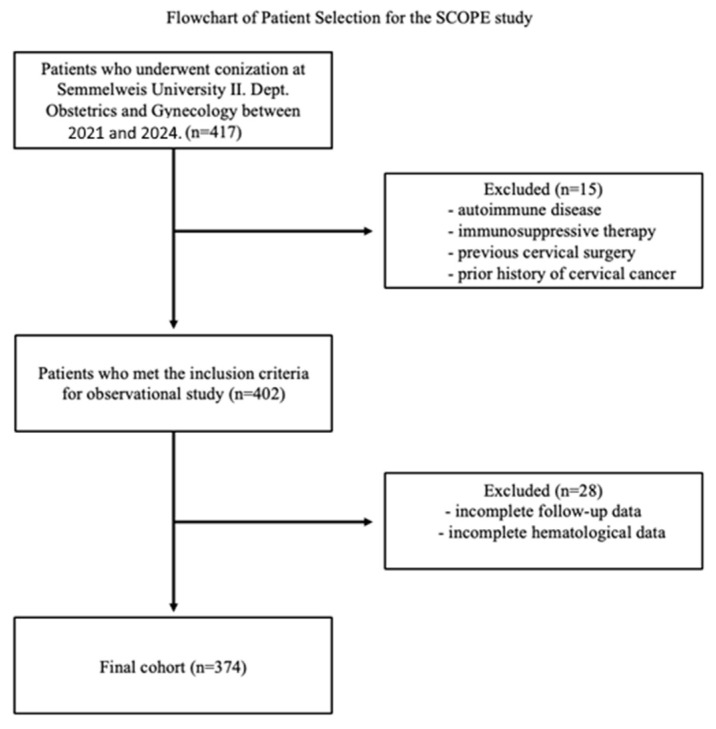
Flowchart of the patient selection. This diagram outlines the stepwise inclusion and exclusion of patients in the study, starting from initial screening through final cohort eligibility based on clinical, cytological, and histological criteria.

Patients who underwent conization at Semmelweis University II. Department of Obstetrics and Gynecology were initially screened (n = 417). Inclusion criteria required patients to have undergone conization for cervical dysplasia and have complete laboratory and clinical data. Patients were excluded if they had a history of autoimmune disease, immunosuppressive therapy, previous cervical surgery, prior cervical cancer diagnosis, or if follow-up or hematological data were incomplete. After applying these criteria, 374 patients were eligible for final analysis. Figure 1 illustrates the patient selection process.

### 2.2. Characteristics

The sociodemographic characteristics included the age of the patients, which was determined by subtracting the year of birth from the year of surgery. Laboratory parameters encompassed inflammation-related biomarkers, such as neutrophil and lymphocyte counts, from which the neutrophil-to-lymphocyte ratio (NLR) was calculated. All laboratory tests were conducted within one month prior to surgery.

The screening for cervical dysplasia included the results of cervical cancer screening and HPV status, with a particular focus on the presence of high-risk HPV. Based on the cervical screening results, patients were categorized into four grades: Grade 1 for negative findings, Grade 2 for LSIL and ASC-US, Grade 3 for HSIL, ASC-H, AGC, and AIS, and Grade 4 for cancer. Similarly, surgical data included the outcomes of conization and histopathological findings from the conization procedure, with conization samples graded according to the same classification: Grade 1 for negative findings, Grade 2 for LSIL and ASC-US, Grade 3 for HSIL, ASC-H, AGC, and AIS, and Grade 4 for cancer. This grading approach was adopted to consolidate cytological and histological categories with similar clinical implications and management strategies, reduce diagnostic heterogeneity, and avoid statistical underrepresentation of smaller subgroups, in line with the recent literature emphasizing overlapping morphologic features and risk-based categorization in cervical cytology interpretation [15].

This study was ethically approved by the Institutional Review Board of Semmelweis University (SE RKEB: 195/2024).

### 2.3. Data Management

For this retrospective analysis, data were gathered and systematically recorded in a specialized database created for the SCOPE study. The database contained detailed patient information, including sociodemographic characteristics, clinical parameters, and laboratory findings. Before conducting the analysis, data quality was meticulously validated through thorough checks for inconsistencies and outliers, employing box plot visualizations to detect extreme values that could impact the results. Any missing data were managed using predefined protocols to maintain the integrity and reliability of the dataset for statistical assessment.

### 2.4. Statistical Analysis

Statistical analyses were conducted using IBM SPSS Statistics for Windows, Version 25.0 (Released 2017, IBM Corp., Armonk, NY, USA). Descriptive statistics (mean, standard deviation, median, and range) were computed for continuous variables, and frequencies and percentages were reported for categorical data to describe the study population. Group comparisons of the neutrophil-to-lymphocyte ratio (NLR) across cytological and histopathological grades were performed using the Kruskal–Wallis test. Post hoc pairwise comparisons were conducted using Dunn–Bonferroni correction to control for multiple testing. For each Kruskal–Wallis test, the effect size was estimated using eta squared (η^2^) to contextualize *p*-values and assess the magnitude of observed differences. To assess the relationship between NLR and histologically confirmed cervical malignancy, both univariable and multivariable binary logistic regression analyses were performed. Binary logistic regression was performed using the ‘Enter’ method in IBM SPSS Statistics (v25). In this method, all selected predictors were entered into the model simultaneously. After testing multiple predictors, only the Neutrophil-to-Lymphocyte Ratio (NLR) remained statistically significant (*p* = 0.008), and therefore it was retained in the final model. No automated stepwise selection procedures (such as Forward LR or Backward LR) were applied, to ensure full control over variable inclusion based on clinical and theoretical relevance. In the multivariable model, potential confounding variables (age, smoking status, HPV DNA positivity) were entered using a forward stepwise (likelihood ratio) method. Odds ratios (ORs) with 95% confidence intervals (CIs) were reported for all predictors retained in the final models. Missing data were addressed using listwise deletion (complete-case analysis) in all regression models. Accordingly, only the cases with complete data for all included variables were retained for multivariate analyses. The number of valid cases (N) for each model is indicated separately. The discriminatory ability of NLR was further evaluated using receiver operating characteristic (ROC) curve analysis. The area under the curve (AUC) was calculated to assess model performance. Optimal NLR cut-off points were determined using the Youden index and Closest Top Left method. Based on these thresholds, sensitivity, specificity, positive and negative predictive values (PPV, NPV), positive and negative likelihood ratios (+LR, −LR), and overall accuracy were computed. Model fit was evaluated using Cox & Snell R^2^ and Nagelkerke R^2^ coefficients. The significance of regression models was tested using omnibus chi-square tests and ANOVA where applicable. Missing values were handled using listwise deletion (complete-case analysis), and the number of valid cases is reported for each statistical procedure. Lastly, a post hoc power analysis was performed using G*Power 3.1.9.7. Based on the observed effect size for NLR (OR = 1.372), alpha = 0.05, and desired power of 0.80, the required minimum sample size was 213. As no prior power calculation was performed due to the retrospective design, we conducted a post hoc power analysis using G*Power (v3.1.9.7). With an observed odds ratio of 1.372 for the NLR variable, α = 0.05, and a desired power of 80%, the estimated minimum sample size was 213. Given our final sample size (n = 374), the study had sufficient power to detect the observed effect. The available sample (n = 374) exceeded this threshold, confirming adequate statistical power to detect the observed association. All statistical tests were two-tailed, and *p*-values < 0.05 were considered statistically significant. This rigorous and transparent statistical approach supports the validity of our findings and ensures proper contextualization of results in terms of both statistical and clinical relevance. This comprehensive approach ensures that the predictive capabilities of NLR in the context of cervical cancer diagnosis and progression are thoroughly examined and that the clinical utility of NLR as a non-invasive biomarker is rigorously evaluated.

## 3. Results

### 3.1. Patient Characteristics

The characteristics of the study participants are summarized in Table 1. Following quality control and outlier exclusion, a total of 373 conization procedures were analyzed. The median age of the patients was 40 years, with an interquartile range of 33 to 47 years. The median body mass index (BMI) was 22.85, ranging from 14.6 to 46.4. Regarding HPV status, 61% of patients were HPV-positive, with high-risk HPV detected in 228 individuals. The median neutrophil-to-lymphocyte ratio (NLR) was 1.85, with a range of 0.47 to 16.86. Cervical cancer screening results categorized patients into four groups: 10 patients (2.7%) were classified as Grade I (negative), 73 (19.5%) as Grade II (LSIL, ASC-US), 274 (73.3%) as Grade III (HSIL, ASC-H, AGC, AIS), and 17 (4.5%) as Grade IV (cancer) (Table 1). Similarly, histopathological findings from conization were classified into four grades: 84 (22.5%) were Grade I (negative), 32 (8.6%) were Grade II (LSIL, ASC-US), 234 (62.3%) were Grade III (HSIL, ASC-H, AGC, AIS), and 23 (6.2%) were Grade IV (cancer) (Table 1).

Table 1: Basic Characteristics Categorical parameters are presented as n. Continuous data are presented as median (range).

### 3.2. Relationship Between Laboratory Parameters and Cytological Results

**Figure 2:**
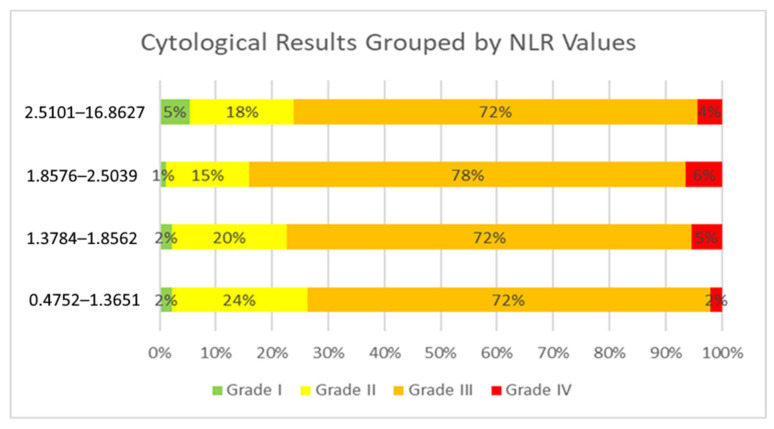
Distribution of cytological results by NLR categories. Stacked bar plots illustrate the proportion of cytology grades stratified by NLR categories. The x-axis represents the percentage of patients, while the color-coded bars indicate negative (green), mild (yellow), severe (orange), and cancerous (red) findings. Higher NLR values are associated with more severe cytological abnormalities.

**Figure 3:**
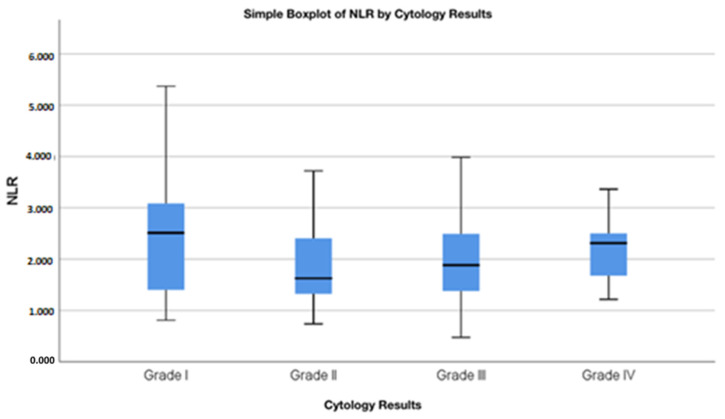
Boxplot of NLR Values by Cytological Outcome. This boxplot illustrates the distribution of neutrophil-to-lymphocyte ratio (NLR) values across different grades of cytological outcome categories. The median and interquartile range are displayed for each group, showing a trend of increasing NLR values with worsening cytological results.

The cytological groups did not show statistically significant differences in NLR values (Kruskal–Wallis test: χ^2^ = 5.785; *p* = 0.123), implying that the NLR does not reliably distinguish between pre-cancerous cytological abnormalities (Table 2). Pairwise Mann–Whitney U tests identified a marginally significant difference between Grade II and Grade IV cytology results (*p* = 0.046), but no other inter-group comparisons reached statistical significance. Specifically, patients with Grade II (LSIL, ASC-US) conization results had a median NLR of 1.63, those with Grade III (HSIL, ASC-H, AGC, AIS) results had a median NLR of 1.88, and patients diagnosed with Grade IV results cancer had an average NLR of 2.31 (Table 3). Although it is impossible to define a precise NLR threshold above which cytological results would change, the distribution of NLR values indicates a clear trend (Figure 2). The visual trend of increasing NLR values with worsening cytological categories aligns with but does not reach statistical significance, reinforcing the conclusion that the NLR is not a strong differentiator of cytology grade (Figure 3—Figure S1: Outliers presented). Higher median NLR values were associated with poorer cytological outcomes. Despite a visual trend of increasing NLR values with more severe cytological outcomes, the Kruskal–Wallis test did not reveal a statistically significant difference (*p* = 0.123), and the overall correlation between the NLR and cytology grades was very weak (Spearman’s rho = 0.047, *p* = 0.364). These findings indicate that the NLR does not significantly correlate with cytological severity and should not be considered a reliable marker for distinguishing between cytological categories.

Table 2 Pairwise comparison of NLR values between cytological groups. Pairwise comparisons of neutrophil-to-lymphocyte ratio (NLR) between cytological severity categories using Mann–Whitney U test values and Dunn–Bonferroni post hoc tests following Kruskal–Wallis analysis. Although a nominally significant difference was found between Grade II and Grade IV (*p* = 0.0466), this did not remain significant after Bonferroni correction. No statistically significant differences were detected between any other cytological categories. The U statistic, z-score, and *p*-values indicate whether significant differences exist between groups. * *p* < 0.05.

This table presents the median neutrophil-to-lymphocyte ratio (NLR) values for different cytological outcomes. The table displays the median, range, minimum, and maximum values for cytology grade. The data indicate a trend of increasing NLR values with worsening cytological results.

Pairwise comparison of NLR values between cytological groups comparing neutrophil-to-lymphocyte ratio (NLR) values between different cytological grade groups.

### 3.3. Relationship Between Laboratory Parameters and Histology Results After Conization

The relationship between conization outcomes and the neutrophil-to-lymphocyte ratio (NLR) was analyzed using the Kruskal–Wallis test, which yielded a statistically significant result (*p* = 0.001). Pairwise comparisons using the Mann–Whitney U test confirmed that NLR values were significantly higher in Grade IV (cancerous) lesions compared to Grade I (*p* < 0.001), Grade II (*p* < 0.001), and Grade III (*p* = 0.001) (Table 4). In order to contextualize statistical significance, effect sizes were calculated. For group comparisons, the Kruskal–Wallis test indicated a negligible effect size in the cytological groups (η^2^ = 0.0075), and a small effect size in the histological groups based on conization (η^2^ = 0.0352).

**Figure 4:**
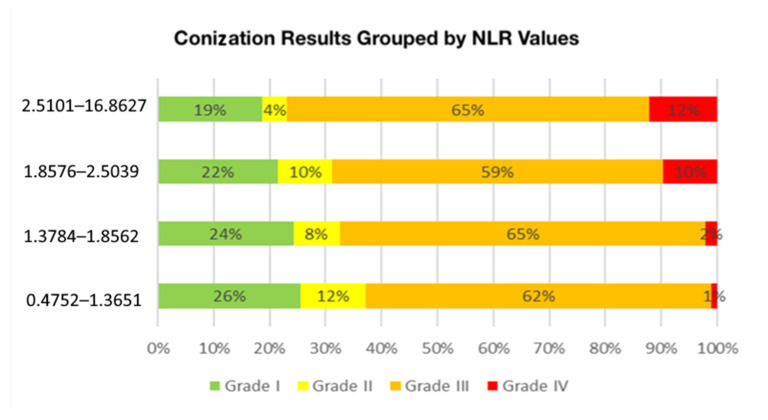
Distribution of Conization Results by NLR Categories Percentage distribution of conization results by histopathological grade is shown across different NLR levels. The x-axis represents the percentage of patients, while the color-coded bars indicate Grade I (green), Grade II (yellow), Grade III (orange), and Grade IV (red) conization findings. Higher NLR values are associated with more severe conization outcomes.

**Figure 5:**
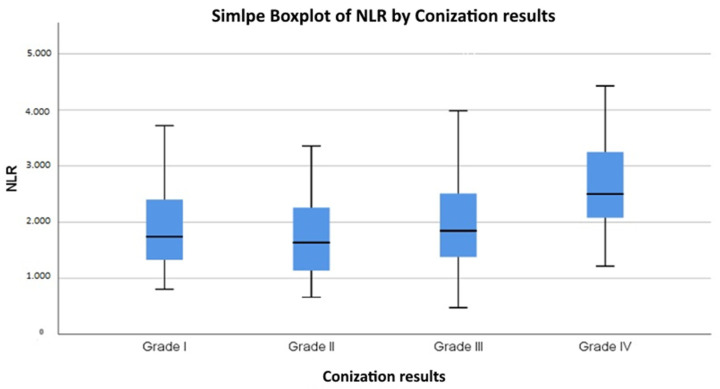
Boxplot of NLR values by conization outcomes.

However, no significant differences were observed between Grades I, II, and III, suggesting that an elevated NLR is primarily associated with malignant conization outcomes. The data indicate that the average NLR value increases proportionally as conization outcomes worsen (Figure 4). Specifically, patients with Grade II (LSIL, ASC-US) conization results had a median NLR of 1.63, those with Grade III (HSIL, ASC-H, AGC, AIS) results had a median NLR of 1.85, and patients diagnosed with Grade IV results cancer had an average NLR of 2.5 (Table 5). A pairwise comparison of NLR values between conization outcome groups and further analysis showed that an NLR value above 2.86 was associated with a higher likelihood of having a cancerous conization outcome (*p* = 0.045) (Figure 5—Figure S2: Outliers presented). Figure 5 also illustrates the spread of NLR values within each histological grade, with Grade IV showing not only a higher median but also wider interquartile ranges compared to Grades I–III. This broader distribution may reflect greater inflammatory variability among patients with confirmed malignancy and further supports the potential relevance of the NLR in identifying high-grade lesions.

This suggests that elevated NLR values may be indicative of worse conization results, reinforcing the potential role of systemic inflammation in cervical dysplasia progression. The correlation between the NLR and histological outcomes was found to be very weak and positive (Spearman’s rho = 0.085, *p* = 0.102), suggesting that as the severity of conization abnormalities increases, the mean NLR values also tend to be elevated.

This boxplot illustrates the distribution of NLR values across different conization outcome categories. The median and interquartile range are displayed for each group, highlighting the trend of increasing NLR values with worsening histopathological results.

To account for potential confounders, we performed a multivariable binary logistic regression analysis using a forward stepwise (likelihood ratio) method. Among the variables assessed (NLR, age, smoking status, HPV DNA positivity), only the NLR and age were retained in the final model. Both variables remained statistically significant predictors of histologically confirmed cervical cancer. The odds ratio (OR) for the NLR was 1.293 (*p* = 0.033), and for age, it was 1.068 (*p* = 0.025), suggesting that increases in these variables are associated with elevated cancer risk (Table 6).

### 3.4. Logistic Regression Model

In logistic regression analyses, the odds ratio for the NLR in the univariable model was 1.372 (95% CI: 1.085–1.735, *p* = 0.008), indicating a moderate increase in risk per unit increase in the NLR (Table 7). In the multivariable model adjusted for age, HPV status, and smoking, the NLR remained an independent predictor (OR = 1.293, 95% CI: 1.021–1.638, *p* = 0.033). These effect size metrics support the conclusion that although some associations are statistically significant, their practical impact should be interpreted with caution.

We applied a logistic regression model to examine the impact of the neutrophil-to-lymphocyte ratio (NLR) laboratory value on the likelihood of a cancer diagnosis (Table 6). The model is overall significant (χ^2^(1) = 8.012, *p* = 0.005), indicating that the NLR variable contributes to the predictive power of the model.

Although the Cox & Snell R^2^ (0.021) and Nagelkerke R^2^ (0.057) coefficients suggest that the NLR explains only a limited proportion of the variance in cancer status, the model is still of interest given its statistically significant association (*p* < 0.05).

From the analysis of the variables, it is evident that the NLR laboratory value is a significant predictor of cancer diagnosis (B = 0.316, S.E. = 0.119, Wald = 7.013, *p* = 0.008). The exp(B) value of 1.372 indicates that a one-unit increase in the NLR laboratory value increases the likelihood of a cancer diagnosis by 37.2% (Table 7).

According to the results of the ANOVA analysis, the regression model is significant (F(1, 371) = 5.950, *p* = 0.015), which reinforces the notion that the NLR laboratory value plays an important role in predicting cancer diagnosis.

Based on the results, it can be concluded that taking the NLR laboratory value into account allows for the prediction of a cancer diagnosis, which could lead to further research and clinical applications. Future studies may aim to explore the interactions between the NLR and other possible predictors, as well as to gain a deeper understanding of its clinical relevance.

This table shows the logistic regression results evaluating NLR as a predictor of cancer diagnosis. The B coefficient (0.316, *p* = 0.008) indicates a significant positive association, with an odds ratio (Exp(B) = 1.372), meaning that each unit increase in NLR raises the cancer risk by 37.2%.

### 3.5. Diagnostic Performance

**Figure 6:**
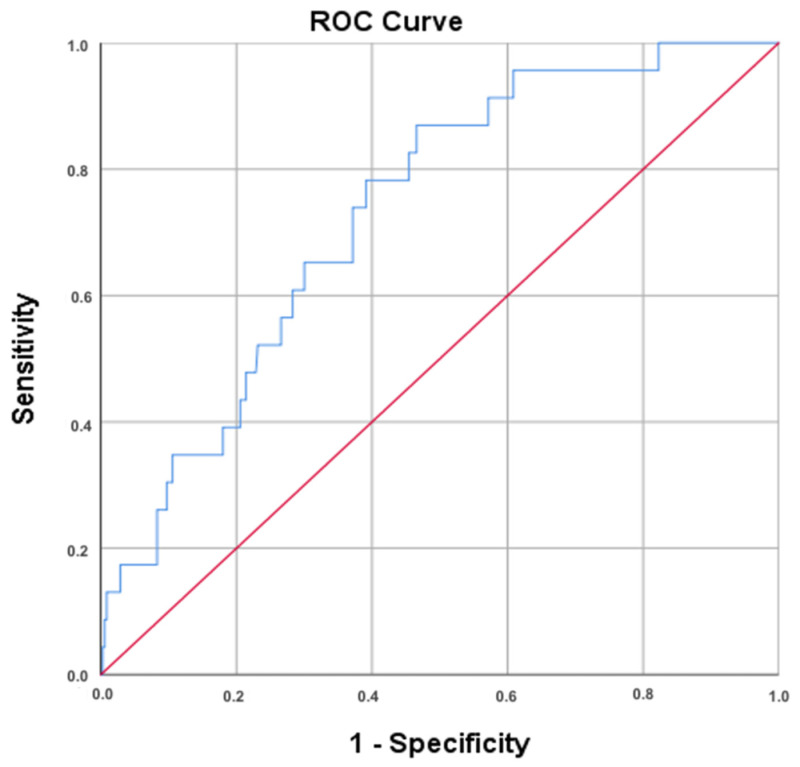
Receiver Operating Characteristic (ROC) Curve for NLR in Predicting cervical cancer. ROC curve compares the diagnostic performance of the neutrophil-to-lymphocyte ratio for identifying cervical cancer. Sensitivity (true positive rate) is plotted on the y-axis, while 1-specificity (false positive rate) is on the x-axis. AUC = 0.734 indicates moderate diagnostic utility.

A receiver operating characteristic (ROC) analysis was performed to assess the diagnostic utility of the NLR in predicting cancer (Figure 6). The area under the curve (AUC) for the NLR was 0.734, indicating a moderate discriminatory ability. The asymptotic 95% confidence interval ranged from 0.643 to 0.825, with a standard error of 0.047 (*p* < 0.001), confirming statistical significance.

For the optimal NLR cut-off determined using the Youden index (≥1.865), the sensitivity was 87.0%, and the specificity was 53.8%. Additionally, using the Closest Top Left method, the best NLR cut-off was ≥ 2.041, yielding a sensitivity of 78.3% and specificity of 61.0%. In addition to the AUC and sensitivity/specificity values derived from the ROC curve, further diagnostic performance metrics were calculated for both NLR thresholds (1.865 and 2.041). These included PPV, NPV, +LR, −LR, and overall accuracy. While the NPV values were high (>97%), the PPV values remained low (<12%), indicating limited utility in confirming disease presence, but potential value in ruling it out (Table 8).

The table shows the different optimal cut-off values with the Youden index and the Closest Top Left method to assess the sensitivity and specificity of the NLR.

## 4. Discussion

The SCOPE study provides evaluation of the neutrophil-to-lymphocyte ratio (NLR) as a predictive biomarker for cervical cancer in patients undergoing LEEP conization. By analyzing the relationship between systemic inflammation and histopathological outcomes, our research findings suggest that higher NLR values are associated with malignant conization results, with an identified NLR threshold ≥2.86 significantly predicting cancer presence (*p* = 0.045). Moreover, logistic regression analysis confirms that each unit increase in the NLR increases the likelihood of a cancer diagnosis by 37.2% (*p* = 0.008).

Cervical cancer remains a significant public health concern, with approximately 604,000 new cases and 342,000 deaths reported annually [16]. Despite WHO’s strategic goal of reducing cervical cancer incidence to fewer than four cases per 100,000 women annually through vaccination, screening, and treatment, substantial gaps persist in early detection and intervention [17]. High-risk patients with abnormal Pap smear results often experience delays in diagnosis, underscoring the need for more effective, accessible biomarkers, particularly in low-resource settings where supplementary tests may not be available due to financial limitations [18].

Chronic inflammation is recognized as a contributing factor in the development of certain cancers, with some estimates suggesting that up to 25% of cases may be associated with prolonged inflammatory conditions [19]. Additionally, tumors not initially caused by inflammation can still elicit an inflammatory response in the surrounding tissue. The systemic inflammation can be manifested as neutrophilia, thrombocytosis, and relative lymphocytopenia detected in a complete blood count (CBC) examination [20]. The neutrophil-to-lymphocyte ratio, derived from simple and cost-effective blood tests, provides critical insights into systemic inflammation and the balance between neutrophils and lymphocytes, both essential for acquired immunity. Previous studies have primarily explored the prognostic implications of an elevated NLR in terms of survival or recurrence. In cervical cancer patients, the NLR has been primarily utilized for follow-up, with higher values being associated with a worse prognosis [21,22,23,24]. Moreover, elevated pretreatment NLR levels have been found to inversely correlate with survival time, further underscoring its potential as a prognostic biomarker in cervical carcinoma. These findings suggest that while the NLR has been extensively investigated as a prognostic and predictive biomarker in several malignancies, including high-grade serous ovarian cancer, and has shown predictive potential in cervical cancer progression, its specific role in predicting malignancy in the context of conization for cervical dysplasia remains insufficiently studied and warrants further investigation [25,26].

Our results revealed a trend of an increasing NLR with the severity of the findings, suggesting an association between systemic inflammation and disease progression. However, the effect size was small, indicating this relationship, while statistically significant, may have limited standalone clinical relevance. The NLR showed a statistically significant association with conization outcomes; moreover, our findings indicate that an NLR threshold of 2.86 significantly predicted cancerous conization results (*p* = 0.045). These results align with the meta-analysis by Zhuang et al., which identified an NLR cut-off value of 3 as a critical prognostic threshold, demonstrating a strong correlation between elevated NLR levels and poorer survival outcomes in cervical cancer patients [27]. While the NLR has shown prognostic value, its association with cytological abnormalities in our study was weak and not statistically significant. The Kruskal–Wallis test (χ^2^ = 5.785, *p* = 0.123) showed no significant differences across cytological grades. However, a Mann–Whitney U test indicated a marginally significant difference between Grade II and Grade IV cytology (*p* = 0.046), suggesting a possible link between higher NLR levels and advanced abnormalities. Despite the lack of a definitive threshold, an increasing trend in NLR values was observed with more severe cytological outcomes. Although high-risk HPV DNA data were available for most patients, the vast majority were high-risk HPV-positive (61%). This limited variability may explain why high-risk HPV status was not retained as a significant independent predictor in our models. Nevertheless, we acknowledge its clinical relevance and incorporated it in adjusted analyses to ensure comprehensive evaluation.

Research on cervical precancer management suggests that a pretreatment NLR is linked to post-conization outcomes. In patients undergoing conization for high-grade CIN, a higher NLR is associated with an increased risk of residual disease or recurrence. Origoni et al. reported that those with an NLR of 2 or higher had a 27.3% recurrence rate, compared to 15.2% in those with an NLR below 2 [28]. Similarly, another cohort study reported that patients with higher NLRs (as well as positive margins and high-risk HPV) had a significantly greater likelihood of post-LEEP recurrence or residual CIN lesions [29].

Building on these findings, receiver operating characteristic (ROC) analysis was conducted to assess the diagnostic utility of the NLR in predicting cervical cancer. The area under the curve (AUC = 0.734) indicated a moderate discriminatory ability, reinforcing the NLR’s relevance in risk assessment. At an optimal NLR cut-off value of ≥1.865 (determined using the Youden index), the model achieved a sensitivity of 87.0% and specificity of 53.8%. Additionally, using the Closest Top Left method, an optimal cut-off of ≥2.041 yielded 78.3% sensitivity and 61.0% specificity, suggesting that the NLR may serve as a supportive parameter in the diagnostic evaluation of cervical cancer.

These findings provide additional evidence that the NLR could be explored further as a predictive biomarker for cervical cancer. Consistent with this, previous studies have demonstrated that elevated NLR levels correlate with worse clinical outcomes in various malignancies. For instance, Shin et al. reported that an increased preoperative NLR was independently associated with reduced recurrence-free survival (RFS), overall survival (OS), and cancer-specific survival (CSS) in colorectal cancer patients undergoing surgery [30].

To evaluate the NLR’s predictive value, we employed a logistic regression model, which demonstrated that the NLR is a significant predictor of cancerous conization outcomes (χ^2^(1) = 8.012, *p* = 0.005). The model’s coefficient and odds ratio suggest that each unit increase in the NLR increases the likelihood of a cancerous outcome by 37.2%. Further supporting this, quantitative analyses using logistic regression in a large cohort of men undergoing prostate biopsy (with PSA levels of 4–10 ng/mL) found that each one-unit increase in the NLR was associated with a 37% higher odds of cancer detection (adjusted OR = 1.372, *p* = 0.038) [31]. However, our model’s explanatory power, as indicated by Cox & Snell R^2^ (0.021) and Nagelkerke R^2^ (0.057) coefficients, was relatively low, suggesting that while the NLR shows some predictive value, additional factors should be integrated for improved predictive accuracy. The NLR alone explains only part of the risk variance; multivariate models perform better when the NLR is combined with clinical and molecular markers. Lee et al. showed that adding the NLR to a prostate cancer risk model modestly but significantly improved discrimination, suggesting the NLR may add value when used alongside other predictors [31]. In cervical CIN, combining the NLR with surgical margin status greatly improved recurrence prediction, with a logistic model achieving an AUC of ~0.87 and higher sensitivity/specificity than the NLR alone [29]. Despite the statistically significant association between the NLR and the presence of malignancy, the logistic regression model demonstrated limited explanatory power, as reflected in the low Cox & Snell R^2^ (0.021) and Nagelkerke R^2^ (0.057) values. These findings indicate that while the NLR may serve as a useful indicator, it explains only a small portion of the variance in conization outcomes. Therefore, the NLR should not be used as a standalone predictor, and its potential role should be considered in combination with other biomarkers (p16 and Ki-67, high-risk HPV genotyping) and clinical parameters.

These findings highlight the NLR’s potential clinical utility as an integrated test as part of a broader strategy to identify high-risk patients, offering a promising, easily accessible biomarker to support cervical cancer risk assessment and early detection strategies.

### 4.1. Strengths and Limitations

One of the major strengths of this study is its large patient number, robust statistical analysis, and incorporation of multiple tests, including the Kruskal–Wallis test, Mann–Whitney U test, and logistic regression modeling. The use of ROC curve analysis further strengthened our findings by quantifying the sensitivity and specificity of the NLR in predicting cervical cancer. A limitation of this study is that the logistic regression model’s explanatory power is relatively low, which suggests that the neutrophil-to-lymphocyte ratio (NLR) is a significant predictor of cancerous conization outcomes. While NLR values tended to increase with disease severity, the 95% confidence intervals for the median NLR values showed substantial overlap between several categories—particularly among intermediate grades. This indicates that, despite statistically significant differences in some comparisons, the separation between groups is not sharp, and the discriminative power of the NLR across all grades remains limited. Future prospective multi-institutional studies should explore diverse populations with the inclusion of additional factors or biomarkers to enhance the predictive accuracy and improve the overall model and to more accurately define the diagnostic and prognostic utility of the NLR.

### 4.2. Implication for Practice

The findings of our study highlight the clinical utility of the NLR, an easily accessible non-invasive biomarker for predicting conization outcomes. Given its affordability and accessibility in routine clinical practice, the NLR could be particularly beneficial in low-resource settings, where access to advanced diagnostic tools may be limited. However, our results also indicate that the NLR alone is not sufficient for comprehensive risk stratification. Future research should focus on developing multimodal predictive models that incorporate systemic inflammatory markers, molecular biomarkers (e.g., p16, Ki-67), and HPV genotyping to enhance diagnostic accuracy. These integrated approaches could enhance early detection and risk stratification, ultimately contributing to more efficient and accessible cervical cancer prevention strategies, particularly in low-income and underserved populations.

## 5. Conclusions

This study identifies an association between an elevated neutrophil-to-lymphocyte ratio (NLR) and malignant conization outcomes, suggesting that the NLR may serve as a promising, easily accessible non-invasive marker worthy of further investigation for cervical cancer risk assessment.

## Figures and Tables

**Table 1:** Characteristics of the sample.

Characteristics (n = 374)	N (Range or %)
Total Median Age (years)	374 40 (23–78)
Median BMI	22.85 (14.6–46.4)
Median NLR	1.85 (0.47–16.86)
HPV positivity	228 (61%)
Hr HPV positivity	228 (61%)
Cytology results	374
Grade I	10 (2.7%)
Grade II	73 (19.5%)
Grade III	274(73.3%)
Grade IV	17 (4.5%)
Conization results	373
Grade I	84 (22.5%)
Grade II	32 (8.6)
Grade III	234 (62.7%)
Grade IV	23 (6.2%)

**Table 2:** Pairwise comparison of NLR values between cytological groups.

Comparison Between Groups	Mann–Whitney U	z	Bonferroni Corrected *p*
Grade I versus Grade II	263.0	−1.427	0.933
Grade I versus Grade III	1077.5	−1.147	1
Grade II versus Grade III	8989.5	−1.328	1
Grade I versus Grade IV	78.0	−0.352	1
Grade II versus Grade IV	427.0	−1.995	0.279
Grade III versus Grade IV	1826.5	−1.493	0.815

**Table 3:** NLR values by cytological outcome.

	Median	N	Range	Minimum	Maximum
Grade I	2.51132	10	4.560	0.811	5.371
Grade II	1.62500	73	9.713	0.738	10.451
Grade III	1.88246	274	10.429	0.475	10.904
Grade IV	2.31022	17	4.575	1.216	5.791
Total	1.85055	374	10.429	0.475	10.904

**Table 4:** Pairwise comparison of NLR values between conization outcome groups.

Comparison Between Groups	Mann–Whitney U	z	Bonferroni Corrected *p*
Grade I versus Grade II	1212.5	−0.812	1
Grade I versus Grade III	9344.5	−0.669	1
Grade II versus Grade III	3200.5	−1.332	1
Grade I versus Grade IV	486.0	−3.640	0.002
Grade II versus Grade IV	147.0	−3.771	0.001
Grade III versus Grade IV	1519.5	−3.444	0.003

Pairwise comparisons of neutrophil-to-lymphocyte ratio (NLR) values between histopathological grades determined by conization, using Dunn–Bonferroni post hoc tests following Kruskal–Wallis analysis. The overall test was statistically significant (χ^2^ = 15.99, *p* = 0.0011), and post hoc analysis revealed that Grade IV differed significantly from both Grade I and Grade II (adjusted *p* < 0.05), suggesting a strong association between higher NLR values and invasive malignancy.

**Table 5:** NLR values by conization outcomes.

	Median	N	Range	Minimum	Maximum
Grade I	1.73973	84	4.566	0.804	5.371
Grade II	1.63434	32	2.695	0.660	3.355
Grade III	1.84531	234	16.387	0.475	16.863
Grade IV	2.50000	23	9.688	1.216	10.904
Total	1.84713	373	16.387	0.475	16.863

This table presents the median, range minimum, and maximum NLR values for different conization outcomes. The results suggest an increasing trend in NLR values with worsening histopathological findings.

**Table 6:** Logistic regression analysis of NLR and age as predictors of malignancy in conization outcomes.

Predictor	B	S.E.	Wald	df	Sig.(p)	Exp(B)	95% CI for Exp(B)
NLR	0.257	0.12	4.565	1	0.033	1.293	[1.021–1.638]
Age	0.066	0.029	5	1	0.025	1.068	[1.008–1.132]

This table presents the results of a multivariate logistic regression analysis. B indicates the unstandardized regression coefficient; S.E., standard error; Wald, Wald chi-square test statistic; df, degrees of freedom; Sig.(p), significance level; Exp(B), odds ratio; 95% CI for Exp(B), 95% confidence interval for the odds ratio.

**Table 7:** Logistic regression analysis of NLR as a predictor of cancer diagnosis.

	B	S.E.	Wald	df	Sig.(p)	Exp(B)	95% CI for Exp(B)
NLR	0.316	0.119	7.013	1	0.008	1.372	[1.085–1.735]
Constant	−3.467	0.372	86.973	1	0.000	0.031	

**Table 8:** Cut-off values via the Youden index and the Closest Top Left method.

	NLR Cut-Off	Sensitivity	1-Specificity	Positive Predictive Value (PPV)	Negative Predictive Value (NPV)	Positive Likelihood Ratio (+LR)	Negative Likelihood Ratio (−LR)	Accuracy
Youden index	1.86510	0.870	0.462	0.109	0.984	1.867	0.244	0.555
Closest Top Left method	2.04119	0.783	0.390	0.116	0.977	1.999	0.357	0.619

## Data Availability

The data supporting the findings of this study can be obtained by contacting the corresponding author upon request.

## Supplementary Materials

The following supporting information can be downloaded at: https://www.mdpi.com/article/10.3390/cancers17111856/s1, Figure S1: Boxplot of NLR Values by Cytological Outcome. This boxplot illustrates the distribution of neutrophil-to-lymphocyte ratio (NLR) values across different cytological outcome grades. The median, interquartile range, and outliers are presented for each group, highlighting a trend of increasing NLR values with worsening cytological results. Outliers are also shown to provide a more comprehensive understanding of data distribution; Figure S2: Boxplot of NLR Values by Conization Outcome. This boxplot illustrates the distribution of neutrophil-to-lymphocyte ratio (NLR) values across different conization outcome grades. The median, interquartile range, and outliers are presented for each group, highlighting a trend of increasing NLR values with worsening conization results. Outliers are also shown to provide a more comprehensive understanding of data distribution.

## Abbreviations

The following abbreviations are used in this manuscript:

AUC	Area Under the Curve
CIN	Cervical Intraepithelial Neoplasia
HPV	Human Papillomavirus
HR-HPV	High-Risk Human Papillomavirus
LEEP	Loop Electrosurgical Excision Procedure
NLR	Neutrophil-to-Lymphocyte Ratio
ROC	Receiver Operating Characteristic
