# Supplementary material for: The Neutrophil-to-Lymphocyte Ratio (NLR) as a Potential Predictor in Conization Outcomes for Cervical Cancer
Source: Cancers (Basel). 2025 May 31;17(11):1856. doi: 10.3390/cancers17111856 (PMC12153780; doi:10.3390/cancers17111856)
Supplement: Supplementary file 1 [file cancers-17-01856-s001.zip › cancers-3627331-Supplementary.pdf]

Supplementary Material

Title: The neutrophil-to-lymphocyte ratio (NLR) as a new predictive factor in cervical cancer

1. **Supplementary Figure S1**
2. **Supplementary Figure S2**

Supplementary Figure S1

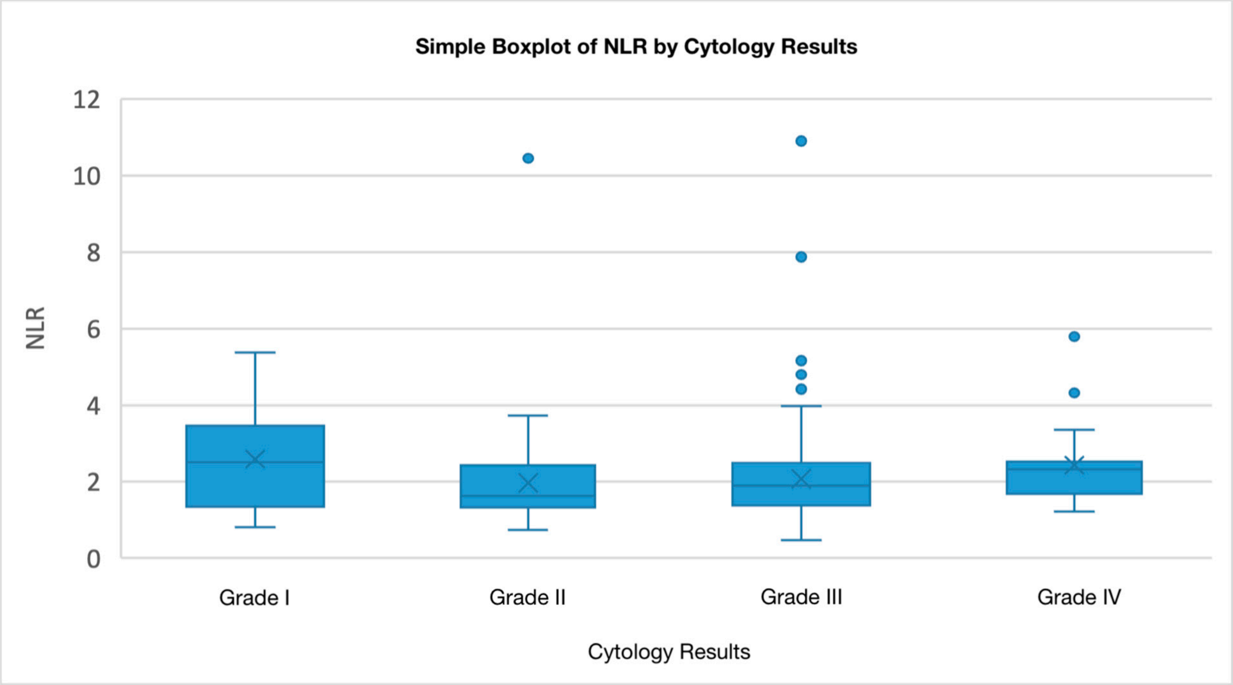

Supplementary Figure S1: Boxplot of NLR Values by Cytological Outcome. This boxplot illustrates the distribution of Neutrophil-to-Lymphocyte Ratio (NLR) values across different cytological outcome grades. The median, interquartile range, and outliers are presented for each group, highlighting a trend of increasing NLR values with worsening cytological results. Outliers are also shown to provide a more comprehensive understanding of data distribution.

Supplementary Figure S2

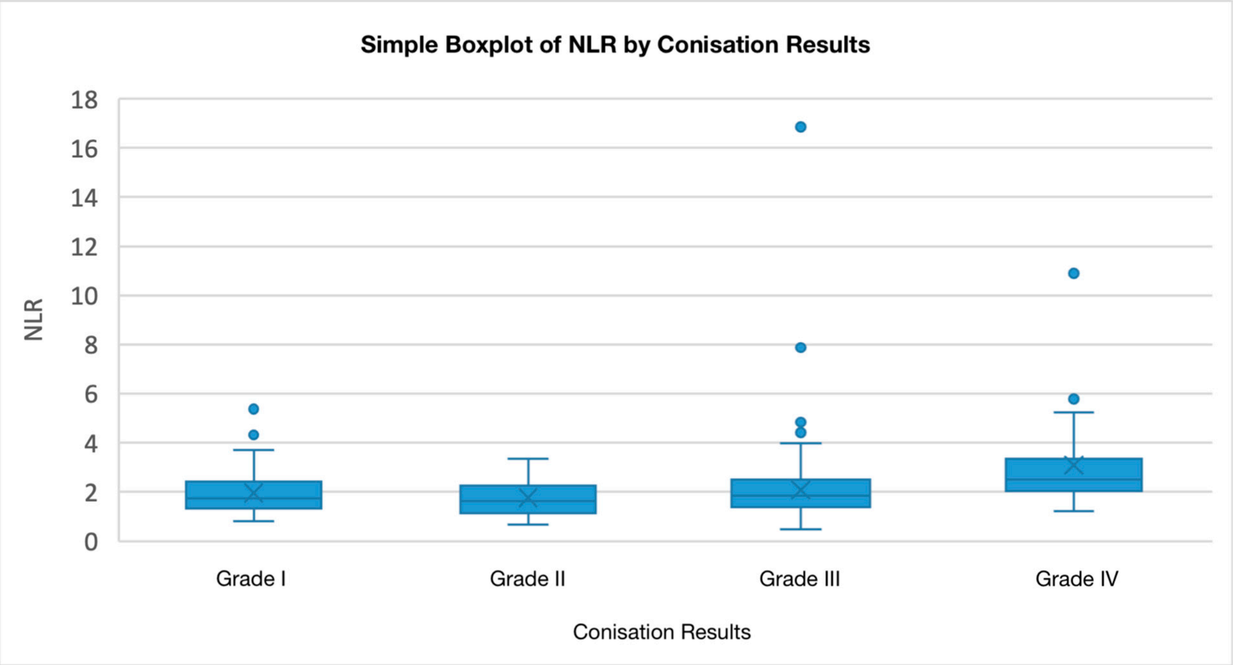

Supplementary Figure S2: Boxplot of NLR Values by Conisation Outcome. This boxplot illustrates the distribution of Neutrophil-to-Lymphocyte Ratio (NLR) values across different conization outcome grades. The median, interquartile range, and outliers are presented for each group, highlighting a trend of increasing NLR values with worsening conization results. Outliers are also shown to provide a more comprehensive understanding of data distribution.
